# Cardiovascular Surgical Emergencies in France, before, during and after the First Lockdown for COVID-19 in 2020: A Comparative Nationwide Retrospective Cohort Study

**DOI:** 10.3390/life11111245

**Published:** 2021-11-16

**Authors:** Anna Baudry, Anne-Sophie Mariet, Eric Benzenine, Valentin Crespy, Chloé Bernard, Marie-Catherine Morgant, Yannick Bejot, Maurice Giroud, Olivier Bouchot, Eric Steinmetz, Catherine Quantin

**Affiliations:** 1 Department of Cardiovascular and Thoracic Surgery, University Hospital of Dijon, 21000 Dijon, France; anna.baudry@chu-dijon.fr (A.B.); valentin.crespy@chu-dijon.fr (V.C.); chloe.bernard@chu-dijon.fr (C.B.); mariecatherine.morgant@chu-dijon.fr (M.-C.M.); olivier.bouchot@chu-dijon.fr (O.B.); eric.steinmetz@chu-dijon.fr (E.S.); 2 Biostatistics and Bioinformatics (DIM), University Hospital of Dijon, 21000 Dijon, France; anne-sophie.mariet@chu-dijon.fr (A.-S.M.); eric.benzenine@chu-dijon.fr (E.B.); 3 CIC1432, University Hospital of Dijon, Clinical Investigation Center, Clinical Epidemiology/Clinical Trials Unit, 21000 Dijon, France; 4 Neurology Department, University Hospital of Dijon, 21000 Dijon, France; yannick.bejot@chu-dijon.fr (Y.B.); maurice.giroud@chu-dijon.fr (M.G.); 5 Dijon Stroke Registry (Santé Publique France–Inserm)-EA 7460 (Pathophysiology and Epidemiology of Cerebro-CardioVascular Diseases), University of Burgundy, UFBC, 21000 Dijon, France; 6 Université Paris-Saclay, UVSQ, University of Paris-Sud, Inserm, High-Dimensional Biostatistics for Drug Safety and Genomics, CESP, 94800 Villejuif, France

**Keywords:** coronavirus disease 2019, hospitalizations, lockdown, cardiovascular emergencies, aortic and arterial diseases, circulatory assistance

## Abstract

Background: There are still gaps regarding the impact of the nationwide lockdown on non-COVID-19 emergency hospitalizations. This study aims to describe the trends in hospitalizations for cardiovascular surgical emergencies in France, before, during and after the first lockdown. Materials and Methods: All adults admitted for mechanical complications of myocardial infarction (MI), aortic dissection, aortic aneurysm rupture, acute and critical limb ischemia, circulatory assistance, heart transplantation and major amputation were included. This retrospective cohort study used the French National Hospital Discharge database. The numbers of hospitalizations per month in 2020 were compared to the previous three years. Results: From January to September 2020, 94,408 cases of the studied conditions were reported versus 103,126 in the same period in 2019 (−8.5%). There was a deep drop in most conditions during the lockdown, except for circulatory assistance, which increased. After the lockdown, mechanical complications of MI and aortic aneurysm rupture increased, and cardiac transplantations declined compared with previous years. Conclusion: We confirmed a deep drop in most cardiovascular surgical emergencies during the lockdown. The post-lockdown period was characterized by a small over-recovery for mechanical complications of MI and aortic aneurysm rupture, suggesting that many patients were able to access surgery after the lockdown.

## 1. Introduction

From a public health policy point of view, we now have a good understanding of the impact of the first nationwide lockdown for coronavirus disease (COVID-19) on hospital admissions for non-COVID-19 medical emergencies [1]. However, the data are still incomplete for medical and surgical cardiovascular emergencies, which are a leading cause of handicap and mortality worldwide [2,3].

Any delay in the management of cardiovascular surgical emergencies, such as ventricular septal rupture or papillary muscle rupture, which are some of the consequences of acute myocardial infarction (MI) [4], critical limb ischemia and secondary amputation [5] and aortic aneurysm ruptures and aortic dissection (AD) [6], can have devastating consequences on survival or functional outcomes. Moreover, discriminating between acute cardiac and COVID-19-induced respiratory distress may be challenging [7].

The COVID-19 pandemic and associated long-term lockdown have led to a severe treatment gap due to the effects on medical practices, the organization of hospital care, as well as in the behavior of patients and clinicians. In developed countries, a significant decrease in emergency room visits [1], surgical emergencies [8], cardiac interventional activity and management of acute coronary syndromes [9,10,11,12] and other emergency vascular activities [13,14,15] has been clearly demonstrated. However, most data were collected during the lockdown period without sufficient follow-up regarding data ascertainment, thus, raising the problem of incomplete data.

Thus far, there have been no complete nationwide studies on major cardiovascular surgical emergencies gathering all hospital admissions in all sizes and types of hospitals. While the magnitude of the initial drop in hospital admissions is well known, we have no data regarding the speed at which admissions returned to previous levels in the post-lockdown period [16].

Therefore, the objective of this study is to describe the nationwide trends in hospital admissions for cardiovascular surgical interventions, including all public and private French hospitals, before, during and after the first lockdown for COVID-19. We focus particularly on the speed at which hospital admissions returned to or surpassed their previous levels during the post-lockdown period in spring 2020.

## 2. Methods

The nationwide data of this retrospective cohort study were provided by the French National Hospital Discharge database (Programme de Médicalisation des Systèmes d’Information, PMSI). This process was approved by the National Committee for Data Protection. While PMSI data are confidential, they are available for researchers who meet specific criteria for access defined by the Agency for Information on Hospital Care (Agence Technique de l’Information sur l’Hospitalisation, ATIH).

### 2.1. Hospitalization Data

Hospitalization data from the same period of time (January to September) over four consecutive years (2017 to 2020) were extracted from the French National Discharge database, which collects the medical records of all patients discharged from all public and private hospitals in France. Hospitalizations included possible hospital transfers.

Diseases were identified according to the International Classification of Diseases-Tenth Revision Codes: aortic dissection (code I71.0); aortic aneurysm rupture (codes I71.1, I71.3, I71.5 and I71.8); acute limb ischemia (codes I74.0, I74.3, I74.4 and I74.5); critical limb ischemia (codes T87.5 and I70.21); mechanical complications of acute MI, i.e., free wall rupture (codes I23.3 and I23.0); ventricular septum rupture (code I23.2); and acute mitral regurgitation due to papillary muscle rupture (codes I23.4 and I34.0).

Surgical procedures were identified using the French Common Classification of Medical Procedures (CCAM) during the hospital stay and included implant of cardiac assist device (Veno-venous (VV) and Veno-arterial (VA) extracorporeal membrane oxygenation (VV-ECMO and VA-ECMO), monoventricular or internal biventricular mechanical circulatory assistance), heart transplantation and transfemoral, transtibial or transmetatarsal amputations.

COVID-19 was identified using specific codes created by the ATIH for this pandemic. The codes can be used for primary diagnoses but also associated and secondary diagnoses in order to ensure that the four diseases are identified even if another severe disease is the primary diagnosis.

Other variables were extracted: age (in continuous form and with four classes), sex and available cardiovascular risk factors (hypertension, diabetes, obesity and atrial fibrillation (AF)).

### 2.2. Study Design

We retrospectively analyzed all patients admitted to every primary and comprehensive public and private hospital in metropolitan France for mechanical complications of myocardial infarction (free wall rupture, rupture of the ventricular septum and acute mitral regurgitation due to rupture of the papillary muscles), aortic dissection, aortic aneurysm rupture, acute limb ischemia, critical limb ischemia and patients requiring cardiac assist device, heart transplantation and major amputation (transfemoral, transtibial and transmetatarsal), between January and September 2020. 

This period included the first peak of the COVID-19 pandemic and the national complete lockdown from 17 March (week 12) to 10 May 2020 (week 19). Hospitalization numbers were compared to the average hospitalizations for the same period of time, month per month, from 2017 to 2019, which provides a more stable basis for comparison than the use of the previous year only, and week by week in 2019 to be more consistent with the dates of the lockdown since it began and ended mid-month (data by week is only available for 2019 and 2020).

Clinical characteristics and in-hospital death were collected for April 2020 and compared to the means in April of 2017 to 2019. April was the only month entirely affected by the lockdown. This retrospective study had no impact on patient care, and all data were anonymous. This study was authorized by the French Data Protection Authority on July 3, 2020 (Registration number: DR-2020-250 on 07/03/2020).

### 2.3. Statistical Analysis

Qualitative variables are presented as frequencies (percentage). Quantitative variables are presented as medians and interquartile ranges (IQR). The different variables analyzed in the cohort of hospitalized patients were compared using univariable and multivariable logistic regression models and the median test (for age in continuous form), between April 2020 and April 2017 to 2019. The change in the number of stays for each disease in 2020 compared with the mean of 2017 to 2019 by month was plotted as smoothed curves using degree 2 spline functions. The threshold for statistical significance was set at <0.05. All analyses were performed using SAS (SAS Institute Inc, Version 9.4, Cary, NC, USA).

### 2.4. Patient and Public Involvement

Patients or the public were not involved in the design, conduct, reporting, or dissemination plans of our research.

## 3. Results

**Figure 1:**
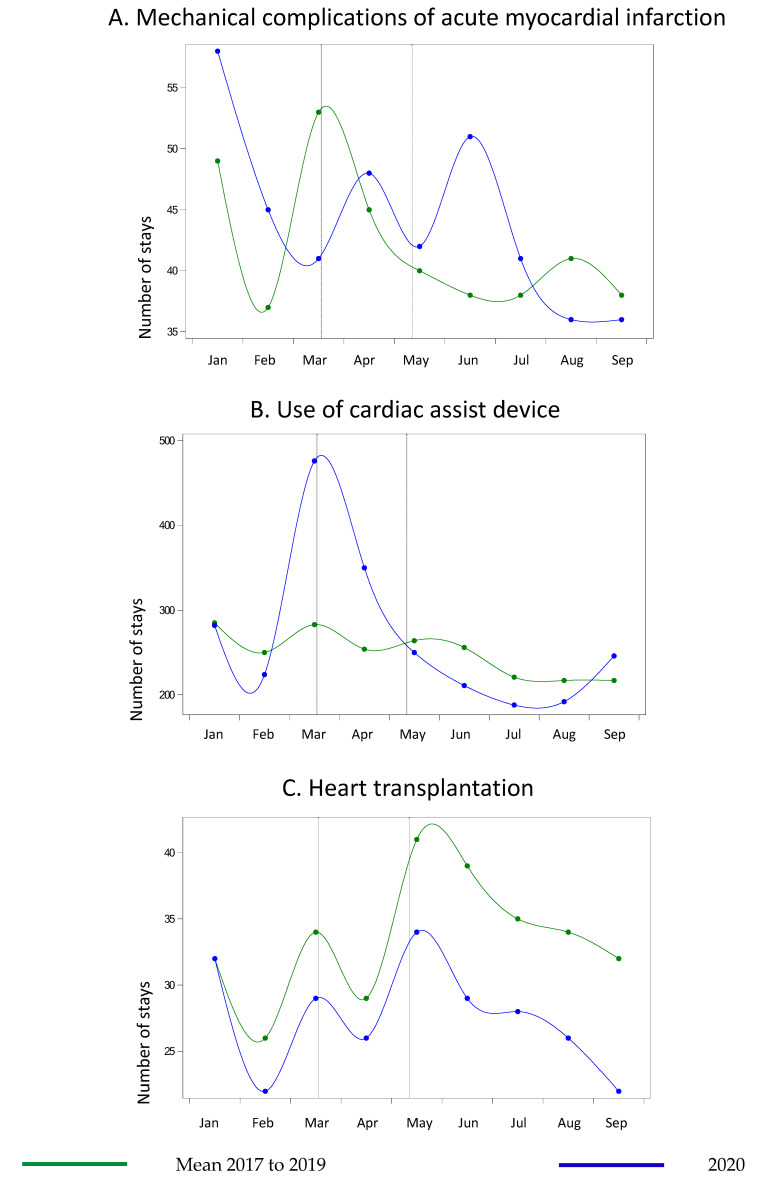
Number of hospitalizations in metropolitan France for cardiac conditions between January and September 2020 compared with the same months from 2017 to 2019. (**A**) Mechanical complications of acute myocardial infarction (**B**) Use of cardiac assist device (**C**) Heart transplantation.

From January to September 2020, 94,408 cases of the cardiovascular diseases and interventions were reported against 103,126 in the same period in 2019, showing an overall decrease in hospital admissions of 8.5% and with a particularly sharp decrease during the lockdown period. There was no change in the number of mechanical complications of MI during lockdown in 2020 (week 12 to 19), but there was an increase of 171.4% (19 vs. 7 patients) right after the end of the lockdown (week 20) (Table 1 and Table S1) (Figure 1A). There was a 62.1% increase in the use of circulatory assistance during the lockdown. 

**Figure 2:**
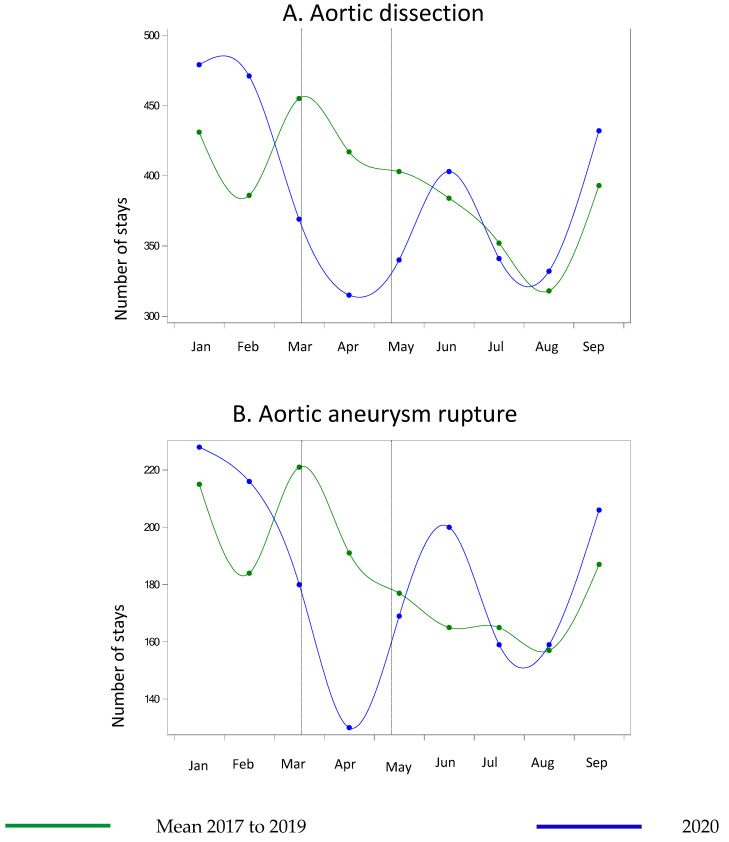
Number of hospitalizations in metropolitan France for aortic conditions between January and September 2020 compared with the same months from 2017 to 2019. (**A**) Aortic dissection (**B**) Aortic aneurysm rupture.

**Figure 3:**
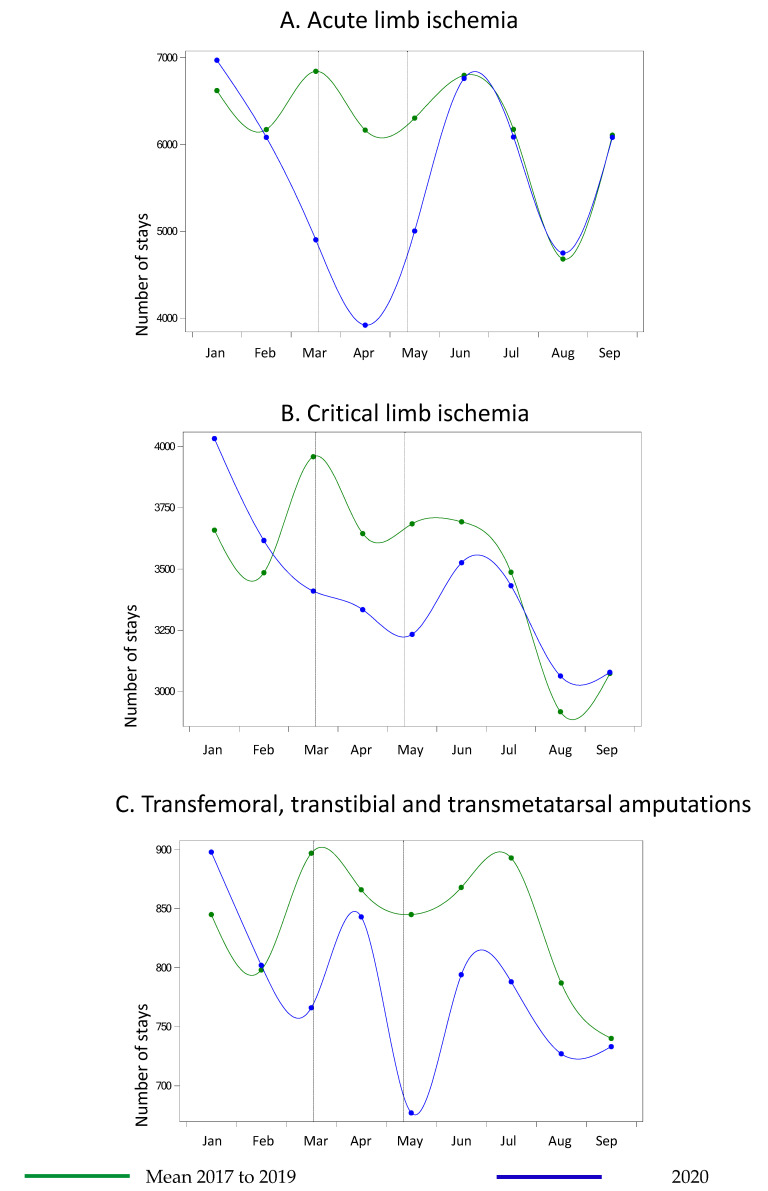
Number of hospitalizations in metropolitan France for vascular peripheral conditions between January and September 2020 compared with the same months from 2017 to 2019. (**A**) Acute limb ischemia (**B**) Critical limb ischemia (**C**) Transfemoral, transtibial and transmetatarsal amputations.

The numbers then began to decrease in the early stage of the lockdown, reaching the trends of previous years before the end of the lockdown period (Figure 1B). Heart transplantations were 16.4% less frequent during the lockdown period. After the end of the lockdown, the variations over time were similar to previous years, but with lower numbers (Figure 1C). For aortic and peripheral vascular diseases, there was a significant drop in the number of hospitalizations that started before the lockdown and gradually returned to the trends seen in previous years after the end of the lockdown period, with maximum variations that varied according to the diseases (Figure 2 and Figure 3) (Table 1 and Table S1). 

The number of AD cases dropped by 33.9% overall, with weekly variations that peaked at −56.1% in week 11, and that did not increase after the end of the lockdown (Figure 2A). The number of aortic aneurysm ruptures decreased by 19.7% and then increased during the month after the end of the lockdown and returned to the previous trends (Figure 2B) in July. The number of acute and critical limb ischemia dropped by between 16.5% (critical) and 39.7% (acute) during the lockdown period (Figure 3A,B). The number of amputations was 7.9% lower during lockdown and remained 8% lower during the post-lockdown period (Figure 3C). The peak weekly variations occurred in week 12 for these three groups, and there was no increase in the number of hospitalizations for these conditions after the end of lockdown (Figure 3).

The clinical characteristics of patients hospitalized for mechanical complications of MI, ruptured aortic aneurysm or amputations were similar between April 2020 and April 2017 to 2019 (Table 2). Patients requiring circulatory assistance in April 2020 were younger (55 vs. 58 years, *p* = 0.01) and had more obesity (34.0% vs. 12.5%, *p* < 0.001) and more hypertension (35.7% vs. 27.0%, *p* = 0.05), and 63.7% were also diagnosed with COVID-19. Patients requiring heart transplantation in April 2020 were more often obese (19.2% vs. 8.1%, *p =* 0.05). 

Patients managed for AD in April 2020 were older (71 vs. 69 years, *p* = 0.0087), had less hypertension (54.0% vs. 59.4%, *p* = 0.03) and atrial fibrillation (18.7% vs. 22.9%, *p* = 0.04), were more often diabetic (13.3% vs. 7.6%, *p* = 0.004) and obese (15.6% vs. 10.4%, *p* = 0.007), and mortality was higher (21.6% vs. 15.4%, *p* = 0.03). For peripheral vascular diseases, patients managed for acute ischemia in April 2020 were more often diabetic (28.8% vs. 25.9%, *p* < 0.001). Mortality was higher for acute ischemia (9.3% vs. 5.8%, *p* < 0.001) and critical ischemia (10.1% vs. 8.4%, *p* = 0.001). COVID-19 was diagnosed in 4.8% to 6.2% of patients with aortic and peripheral vascular diseases.

## 4. Discussion

To our knowledge, this is the first nationwide population-based retrospective cohort study reporting hospitalization numbers for aortic and peripheral vascular surgery and some cardiac surgical interventions in all public and private facilities, before, during and 4 months after the first lockdown for COVID-19. We found a deep drop in activity and diagnoses parallel to the national lockdown. In addition, there was no major “catch-up” after the end of this period.

Nevertheless, major differences were observed: an increase in the mechanical complications of acute MI and aortic aneurysm rupture after the end of lockdown period, a major increase in circulatory assistance use during the peak of the epidemic and an ongoing decline in cardiac transplantations. The overall decrease of 8.5% seems low, but it should be kept in mind that this figure includes the post-lockdown period as well as the spike in the use of circulatory support during the start of the pandemic. Our data for the lockdown period are consistent with data already published in other countries and for other emergencies requiring prompt care [1,8,12,17,18].

We observed a decrease in the hospitalization numbers for AD similar to the literature [19,20], which may have several possible explanations [16]. During this period, the health authorities recommended that the general public stay home to avoid the risk of COVID-19 contamination and to give priority to patients with severe COVID-19. There may have been misdiagnoses and longer times between the first symptoms and hospitalization. People were required to stay at home, resulting in reduced physical activity, effort and stress and, therefore, fewer hypertensive crises, which is the main risk factor for AD and aortic aneurysm rupture. Comparisons with previous years allowed us to rule out the potential seasonal effect of atmospheric pressure on the occurrence of AD or aneurysm ruptures [21].

Regarding cardiac transplantations, the variations over time followed the same patterns in 2019 and 2020, but the number of transplantations remained lower in 2020. This trend is probably due to unknown viral status in potential donors and the lack of clear guidelines for cardiac transplantation management at the beginning of the pandemic. In the literature, there is a case of a recipient rejecting a graft because of concerns about isolation during the recovery period and contamination [22]. The number of organ donors also decreased during the lockdown because there were fewer traffic accidents and a decrease in trauma and brain death [23].

The greatest decrease in admissions was observed for acute ischemia. Its management is a challenge, with a mortality rate of 9% in-hospital and 40% at one year; the amputation rate is between 6% and 9% in-hospital and between 11% and 15% at one year [24,25]. These trends are for treated acute ischemic events, and it can be assumed that they would be much higher without management. For critical ischemia, the decrease was less significant but lasted longer, and the 2020 figures did not reach those of previous years until July. A limitation of our data is the lack of data on severity. Patients presenting with critical ischemia often have co-morbidities, consulting at already advanced stages of the disease, and the stages on arrival were potentially more severe [14,15]. The drop in the number of diagnoses of these pathologies is likely related to inadequate patient management (through changes in patient behavior and hospital organization), rather than to a real decrease in their incidence.

We observed a peak in hospitalizations with circulatory assistance. We could not differentiate the type of assistance between veno-venous and veno-arterial with the existing codes, but this increase is most likely due to the increase in VV-ECMO used to short-term assistance for symptomatic treatment of COVID-19 infection, which was the case for 63.7% of patients requiring cardiac assistance in April 2020. Indeed, the high affinity of SARS-CoV-2 for the angiotensin I converting enzyme 2 receptor helps the virus enter the myocardium and artery vessels, leading to cardiovascular decompensation of pre-existing cardiac conditions [26] that may require circulatory assistance. These patients were younger than in previous years. This may be explained by the differences in indications related to COVID-19 and also by the allocation to patients more likely to benefit from circulatory assistance, given the high level of technical and human resources required to manage this device [27].

One of the major interests this study is the analysis of post-lockdown data, which allowed us to observe the variations in emergency hospitalizations in the four months following the end of lockdown. For aortic aneurysm ruptures, there was a moderate rebound effect in June, which could correspond to a resumption of activity and an increased risk of rupture, or to aneurysms for which surgery had to be postponed during the lockdown. Indeed, there is a demonstrated increased risk of aortic aneurysm mortality due to 3 months of deferred surgery, which may correspond to our observation [28].

The mechanical complications of acute MI must be interpreted while considering the variations in MI incidence. We observed a decrease in MI hospitalizations during the lockdown, likely due to under-diagnosis, under-management and delays in revascularization treatment [11,29]. Additionally, a drop in the incidence of these diseases is possibly due in part to the decrease in air pollution during the lockdown, which is a powerful risk factor for severe cardiovascular diseases [11]. Longer times between the beginning of symptoms and hospital admission leads to a higher risk of ischemic lesions as described previously [30]. Those tendencies may explain the rebound in the number of mechanical complications during the post-lockdown period.

Regarding the other cardiovascular conditions and interventions, we were able to show that, after the lockdown, the hospitalization rates returned to those observed in previous years at the same periods. The fact that there was not an increase in admissions may suggest more deaths of unmanaged patients at home during the lockdown [31]. Our results seem to show that despite the persistence of the global pandemic, for most of these conditions and procedures, the numbers returned to normal after the end of the lockdown, which was likely made possible by adjustments that were made in the health care system and emergency and surgical services in view of the global context.

Regarding the clinical characteristics, patients presented the classical vascular profile for most of the diseases and interventions [5]. It is important to note that, among patients treated for AD and acute and critical ischemia, hospital mortality was higher, which supports the hypothesis of a delay in treatment, either because of postponed consultations, consultation at advanced stages, or a lack of technical and human resources at that time.

These findings highlight the importance of offering the best possible care and follow-up to patients with diseases other than COVID-19. The pandemic period may be not over. Public health recommendations regarding the importance of early management of several acute non-COVID-19 emergencies should be underscored, and the general population needs to be reassured that all the necessary precautions are being taken in emergency rooms and hospitals to ensure patient safety.

One of the limitations of the present study is that it is an observational, descriptive, retrospective study that does not provide precise evaluations of disease severity. A potential misclassification-related or under-detection-related bias may have occurred, especially for comorbidities. Even so, this misclassification bias is likely to be non-differential for the majority of the comorbidities, seeing as this bias may have affected the data collection in the same way during the previous years. Under-detection is possible but likely minimal, particularly for procedures after the lockdown, due to the long post-lockdown period studied, resulting in more complete data capture. Another limitation is the restriction to hospital data, meaning we did not have access to out-of-hospital mortality and its causes and see if there was an increase in out-of-hospital deaths in individuals who were likely unable or unwilling to access care.

The present study has several strengths. To our knowledge, this is the first study of the post-lockdown period for cardiac interventional activity and other emergency vascular activity. Administrative data collection may be hindered by delays in data collection, but we followed up for data collection until December 2020, strengthening the reliability of the data collected over the 4 months after lockdown (until September). All French primary and comprehensive public and private hospital data were included; therefore, the data is nationally exhaustive. The possible influence of a seasonal effect was also reduced by comparing data from 2020 with data from the three previous years for each month. We were thus able to estimate the consequences of the global pandemic and the national lockdown on vascular procedures and patients as recommended by several large international studies [32,33].

## 5. Conclusions

This study provides the first description of French nationwide data from more than 90,000 hospital admissions and surgical interventions for cardiac surgery and other emergency vascular activity during the first complete lockdown and post-lockdown periods for COVID-19. We report, similar to other studies, a severe drop in hospitalization volumes during the lockdown, possibly related to changes in the behavior of patients and clinicians, misdiagnosis and confusion with COVID-19, increased delays between the first symptoms and hospitalization and excess of out-of-hospital mortality. However, we report novel information regarding the recovery in admissions, which did not exceed the prior levels except for aortic aneurysm rupture and mechanical complications of MI.

Our data suggest that many patients did not access acute care and secondary prevention, even if hospitals were able to catch-up on the interventions required for a considerable proportion of these patients at risk of death or complications. Considering that the COVID-19 pandemic is ongoing and that a new lockdown might be still required, this phenomenon should be considered carefully in order to provide appropriate public health messages and to ensure suitable follow-up, particularly for patients with conditions other than COVID-19.

## Figures and Tables

**Table 1:** Variations in the number of hospitalizations for cardiac, aortic and peripheral vascular conditions before, during and after the lockdown in 2020 compared with the same periods in 2019 in France and the week of maximum variation.

	Before Lockdown			During Lockdown			After Lockdown			Maximal Variation	
	2019	2020	Variation	2019	2020	Variation	2019	2020	Variation	%	Week
Cardiac conditions											
Complications of myocardial infarction	104	108	3.8%	83	83	0.0%	200	194	−3.0%	171.4%	20
Use of circulatory assistance	651	634	−2.6%	462	749	62.1%	1036	961	−7.2%	138.8%	13
Heart transplantation	69	68	−1.4%	61	51	−16.4%	165	123	−25.5%	−83.3%	37
Aortic conditions											
Aortic dissection	984	1123	14.1%	841	556	−33.9%	1767	1673	−5.3%	−56.1%	11
Aortic aneurysm rupture	475	516	8.6%	330	265	−19.7%	782	808	3.3%	−42.5%	14
Peripheral vascular conditions											
Acute limb ischemia	15,828	15,348	−3.0%	11,729	7072	−39.7%	27,479	26,580	−3.3%	−50.7%	12
Critical limb ischemia	9154	8991	−1.8%	7006	5853	−16.5%	15,742	14,985	−4.8%	−34.5%	12
Amputations	2012	1955	−2.8%	1563	1439	−7.9%	3732	3435	−8.0%	−25.9%	12

Before lockdown: week 2 to 11. During lockdown: week 12 to 19. After lockdown: week 20 to 39.

**Table 2:** Clinical characteristics of patients in each condition subgroup, with a comparison between April 2020 and April 2017 to 2019 in France.

	2017–2019 N (%) or Median [IQR]	2020 N (%) or Median [IQR]	*p* Value Univariable Logistic Regression ^a^	*p* Value Multivariable Logistic Regression
Complications of myocardial infarction	N = 137	N = 48		
Age (years): continuous	71 (60–83)	67.5 (57.5–77.5)	0.05 ^a^	
Classes:			0.50	0.65
[18–65]	48 (35.0)	16 (33.3)		
[65–80]	45 (32.9)	20 (41.7)		
[80–85]	18 (13.1)	7 (14.6)		
≥85	26 (19.0)	5 (10.4)		
Male	78 (56.9)	33 (68.8)	0.15	0.21
Hypertension	50 (36.5)	21 (43.8)	0.37	0.23
Diabetes	20 (14.6)	4 (8.3)	0.27	0.23
Obesity	9 (6.6)	3 (6.3)	0.94	0.93
Atrial fibrillation	39 (28.5)	10 (20.8)	0.30	0.36
COVID-19	0 (0)	4 (8.3)	NA	NA
Death	66 (48.2)	22 (45.8)	0.78	0.99
Use of circulatory assistance	N = 763	N = 350		
Age (years): continuous	58 (47–66)	55 (46–62)	0.01 ^a^	
Classes:			<0.001	<0.001
[18–65]	159 (20.8)	73 (20.9)		
[65–80]	157 (20.6)	95 (27.1)		
[80–85]	227 (29.8)	128 (36.6)		
≥85	220 (28.8)	54 (15.4)		
Male	564 (73.9)	266 (76.0)	0.46	0.18
Hypertension	206 (27.0)	125 (35.7)	0.003	0.05
Diabetes	148 (19.4)	77 (22.0)	0.32	0.33
Obesity	95 (12.5)	119 (34.0)	<0.001	<0.001
Atrial fibrillation	209 (27.4)	65 (18.6)	0.002	<0.001
COVID-19	0 (0)	223 (63.7)	NA	NA
Death	426 (55.8)	167 (47.7)	0.01	0.10
Heart transplantation	N = 87	N = 26		
Age (years): continuous	54 (43–61)	52.5 (43–56)	0.40 ^a^	
Classes:			0.15	0.15
[18–65]	30 (34.5)	8 (30.8)		
[65–80]	15 (17.2)	9 (34.6)		
[80–85]	12 (13.8)	5 (19.2)		
≥85	30 (34.5)	4 (15.4)		
Male	64 (73.6)	17 (65.4)	0.42	0.31
Hypertension	24 (27.6)	5 (19.2)	0.39	0.20
Diabetes	19 (21.8)	7 (26.9)	0.59	0.92
Obesity	7 (8.1)	5 (19.2)	0.11	0.05
Atrial fibrillation	29 (33.3)	9 (34.6)	0.90	0.89
COVID-19	0 (0)	1 (3.9)	NA	NA
Death	19 (21.8)	5 (19.2)	0.78	0.71
Aortic dissection	N = 1251	N = 315		
Age (years): continuous	69 (59–79)	71 (60–81)	0.009 ^a^	
Classes:			0.05	0.03
[18–65]	485 (38.8)	106 (33.6)		
[65–80]	457 (36.5)	116 (36.8)		
[80–85]	128 (10.2)	49 (15.6)		
≥85	181 (14.5)	44 (14.0)		
Male	794 (63.5)	217 (68.9)	0.07	0.06
Hypertension	743 (59.4)	170 (54.0)	0.08	0.03
Diabetes	95 (7.6)	42 (13.3)	0.002	0.004
Obesity	130 (10.4)	49 (15.6)	0.01	0.007
Atrial fibrillation	286 (22.9)	59 (18.7)	0.11	0.04
COVID-19	0 (0)	15 (4.8)	NA	NA
Death	192 (15.4)	68 (21.6)	0.008	0.03
Aortic aneurysm rupture	N = 574	N = 130		
Age (years): continuous	75 (65–84)	75 (68–84)	0.73 ^a^	
Classes:			0.13	0.15
[18–65]	139 (24.2)	21 (16.2)		
[65–80]	202 (35.2)	58 (44.6)		
[80–85]	93 (16.2)	19 (14.6)		
≥85	140 (24.4)	32 (24.6)		
Male	434 (75.6)	97 (74.6)	0.81	0.72
Hypertension	265 (46.2)	61 (46.9)	0.88	0.55
Diabetes	56 (9.8)	14 (10.8)	0.73	0.80
Obesity	44 (7.7)	6 (4.6)	0.23	0.26
Atrial fibrillation	120 (20.9)	24 (18.5)	0.53	0.48
COVID-19	0 (0)	8 (6.2)	NA	NA
Death	203 (35.4)	54 (41.5)	0.18	0.25
Acute limb ischemia	N = 18,496	N = 3919		
Age (years): continuous	71 (62–81)	72 (63–83)	<0.001 ^a^	
Classes:			<0.001	0.01
[18–65]	5938 (32.1)	1131 (28.9)		
[65–80]	7204 (39.0)	1527 (38.9)		
[80–85]	2165 (11.7)	478 (12.2)		
≥85	3189 (17.2)	783 (20.0)		
Male	12,842 (69.4)	2669 (68.1)	0.10	0.74
Hypertension	8094 (43.8)	1641 (41.9)	0.03	<0.001
Diabetes	4786 (25.9)	1127 (28.8)	<0.001	<0.001
Obesity	1567 (8.5)	298 (7.6)	0.07	0.14
Atrial fibrillation	2565 (13.9)	629 (16.1)	<0.001	0.13
COVID-19	0 (0)	198 (5.1)	NA	NA
Death	1073 (5.8)	366 (9.3)	<0.001	<0.001
Critical limb ischemia	N = 10,934	N = 3334		
Age (years): continuous	77 (67–85)	76 (67–85)	0.02 ^a^	
Classes:			0.13	0.24
[18–65]	2122 (19.4)	632 (19.0)		
[65–80]	4089 (37.4)	1322 (39.6)		
[80–85]	1754 (16.0)	507 (15.2)		
≥85	2969 (27.2)	873 (26.2)		
Male	7465 (68.3)	2331 (69.9)	0.07	0.16
Hypertension	5542 (50.7)	1762 (50.2)	0.59	0.24
Diabetes	5387 (49.3)	1700 (51.0)	0.08	0.23
Obesity	948 (8.7)	333 (10.0)	0.02	0.03
Atrial fibrillation	2460 (22.5)	774 (23.2)	0.38	0.51
COVID–19	0 (0)	177 (5.3)	NA	NA
Death	915 (8.4)	336 (10.1)	0.002	0.001
Amputations	N = 2600	N = 843		
Age (years): continuous	72 (63–82)	73 (64–82)	0.04 ^a^	
Classes:			0.49	0.41
[18–5]	736 (28.3)	226 (26.8)		
[65–80]	1054 (40.5)	351 (41.6)		
[80–85]	372 (14.3)	110 (13.1)		
≥85	438 (16.9)	156 (18.5)		
Male	1955 (75.2)	643 (76.3)	0.53	0.43
Hypertension	1218 (46.9)	399 (47.3)	0.81	0.80
Diabetes	1422 (54.7)	472 (56.0)	0.51	0.62
Obesity	244 (9.4)	94 (11.2)	0.13	0.13
Atrial fibrillation	529 (20.4)	177 (21.0)	0.68	0.99
COVID-19	0 (0)	49 (5.8)	NA	NA
Death	221 (8.5)	79 (9.4)	0.44	0.45

N: number; %: proportion; IQR: interquartile range; NA: not applicable. ^a^ or *p*-value of median test for age in continuous form.

## Data Availability

The PMSI database was transmitted by the national agency for the management of hospitalization data. The use of these data by our department was approved by the National Committee for data protection. We are not allowed to transmit these data. PMSI data are available for researchers who meet the criteria for access to these French confidential data (this access is submitted to the approval of the National Committee for data protection) from the national agency for the management of hospitalization (ATIH—Agence technique de l’information sur l’hospitalisation). Address: Agence technique de l’information sur l’hospitalisation, 117 boulevard Marius Vivier Merle, 69329 Lyon Cedex 03.

## Supplementary Materials

The following are available online at https://www.mdpi.com/article/10.3390/life11111245/s1, Table S1: Monthly hospitalizations for cardiovascular surgical emergencies in France from January to September 2017 to 2019 (mean) and 2020.
