# Supplementary material for: Cardiovascular Surgical Emergencies in France, before, during and after the First Lockdown for COVID-19 in 2020: A Comparative Nationwide Retrospective Cohort Study
Source: Life (Basel). 2021 Nov 16;11(11):1245. doi: 10.3390/life11111245 (PMC8620591; doi:10.3390/life11111245)
Supplement: Supplementary file 1 [file life-11-01245-s001.zip › life-1432722-supplementary.pdf]

life

MDPI

Supplementary Material

Cardiovascular Surgical Emergencies in France, before, during and after the First Lockdown for COVID-19 in 2020: A Comparative Nationwide Retrospective Cohort Study

Table S1. Monthly hospitalizations for cardiovascular surgical emergencies in France from January to September 2017 to 2019 (mean) and 2020.

	Year	January	February	March	April	May	June	July	August	September
Complications of myocardial infarction	2017–2019*	50 (9.4)	37 (4.3)	54 (6.1)	46 (2.6)	40 (2.1)	38 (7.5)	39 (2.5)	42 (1.9)	39 (5.0)
	2020	58	45	41	48	42	51	41	36	36
Use of circulatory assistance	2017–2019*	285 (24.1)	251 (10.0)	284 (12.3)	254 (18.3)	264 (13.6)	256 (4.2)	222 (21.1)	217 (19.6)	217 (10.2)
	2020	282	224	476	350	250	211	188	192	246
Heart transplantation	2017–2019*	33 (1.3)	26 (1.7)	34 (4.9)	29 (2.9)	41 (5.7)	39 (6.9)	35 (5.4)	35 (6.6)	33 (3.9)
	2020	32	22	29	26	34	29	28	26	22
Aortic dissection	2017–2019*	431 (34.7)	386 (16.2)	455 (21.1)	417 (22.2)	403 (31.3)	385 (37.1)	352 (12.3)	318 (5.7)	393 (39.6)
	2020	479	471	369	315	340	403	341	332	432
Aortic aneurysm rupture	2017–2019*	215 (7.8)	184 (12.0)	221 (19.4)	191 (6.0)	177 (12.0)	165 (7.9)	165 (11.2)	157 (10.3)	187 (2.2)
	2020	228	216	180	130	169	200	159	159	206
Acute limb ischemia	2017–2019*	6621 (448.4)	6172 (83.2)	6844 (106.8)	6165 (165.9)	6304 (211.5)	6796 (219.2)	6174 (47.4)	4681 (179.5)	6107 (200.8)
	2020	6970	6082	4902	3919	5003	6761	6086	4749	6082
Critical limb ischemia	2017–2019*	3659 (367.0)	3486 (78.3)	3958 (44.5)	3645 (182.7)	3684 (85.5)	3693 (131.9)	3487 (170.4)	2917 (75.8)	3073 (171.4)
	2020	4032	3616	3410	3334	3233	3526	3432	3063	3078
Amputations	2017–2019*	845 (55.8)	798 (18.9)	897 (42.9)	867 (6.9)	845 (8.5)	869 (39.7)	893 (18.9)	787 (28.3)	740 (46.3)
	2020	898	802	766	843	677	794	788	727	733

* Mean of monthly hospitalization numbers for 2017 to 2019 (standard deviation)
